# Clinical and Radiological Parameters to Discriminate Tuberculous Peritonitis and Peritoneal Carcinomatosis

**DOI:** 10.3390/diagnostics13203206

**Published:** 2023-10-13

**Authors:** Daya K. Jha, Pankaj Gupta, Pardhu B. Neelam, Rajender Kumar, Venkata S. Krishnaraju, Manish Rohilla, Ajay S. Prasad, Usha Dutta, Vishal Sharma

**Affiliations:** 1 Department of Gastroenterology, Postgraduate Institute of Medical Education and Research, Chandigarh 160012, India; dayakrishna.jha@gmail.com (D.K.J.); drpardhu.bharath@gmail.com (P.B.N.); ushadutta@gmail.com (U.D.); 2 Department of Radiodiagnosis, Postgraduate Institute of Medical Education and Research, Chandigarh 160012, India; pankajgupta959@gmail.com; 3 Department of Nuclear Medicine, Postgraduate Institute of Medical Education and Research, Chandigarh 160012, India; drrajender2010@gmail.com (R.K.); venkat.hc@gmail.com (V.S.K.); 4 Department of Cytopathology, Postgraduate Institute of Medical Education and Research, Chandigarh 160012, India; rohillamanishpgi@gmail.com; 5 Department of Gastroenterology, Army Hospital Research and Referral, New Delhi 110010, India; drasprasad2003@yahoo.co.in

**Keywords:** tuberculous peritonitis, abdominal tuberculosis, peritoneal tuberculosis, computed tomography, ascites, malignant ascites, peritoneum, peritoneal carcinomatosis

## Abstract

It is challenging to differentiate between tuberculous peritonitis and peritoneal carcinomatosis due to their insidious nature and intersecting symptoms. Computed tomography (CT) is the modality of choice in evaluating diffuse peritoneal disease. We conducted an ambispective analysis of patients suspected as having tuberculous peritonitis or peritoneal tuberculosis between Jan 2020 to Dec 2021. The study aimed to identify the clinical and radiological features differentiating the two entities. We included 44 cases of tuberculous peritonitis and 45 cases of peritoneal carcinomatosis, with a median age of 31.5 (23.5–40) and 52 (46–61) years, respectively (*p* ≤ 0.001). Fever, past history of tuberculosis, and loss of weight were significantly associated with tuberculous peritonitis (*p* ≤ 0.001, *p* = 0.038 and *p* = 0.001). Pain in the abdomen and history of malignancy were significantly associated with peritoneal carcinomatosis (*p* = 0.038 and *p* ≤ 0.001). Ascites was the most common radiological finding. Loculated ascites, splenomegaly and conglomeration of lymph nodes predicted tuberculous peritonitis significantly (*p* ≤ 0.001, *p* = 0.010, *p* = 0.038). Focal liver lesion(s) and nodular omental involvement were significantly associated with peritoneal carcinomatosis (*p* = 0.011, *p* = 0.029). The use of clinical features in conjunction with radiological findings provide better diagnostic yields because of overlapping imaging findings.

## 1. Introduction

Tuberculosis (TB) is an ancestral and stubbornly prevalent global infection, affecting a quarter of the world’s population [1]. TB affects nearly 10 million people and leads to death in more than a million people annually, which might be just the tip of the iceberg [2]. It remains a global problem with the increasing use of biologics, HIV infection, emigration, and an aging population [3]. TB primarily affects the lungs but nearly 15% of immunocompetent patients and up to 50% of immunocompromised patients can develop extrapulmonary clinical manifestations [4]. Abdominal TB accounts for approximately 15% of extrapulmonary tuberculosis (EPTB) cases with a greater incidence in high TB-burden countries [5]. Abdominal TB can involve all organs but is predominantly limited to the peritoneum, intestine, solid viscera, and lymph nodes. Tuberculous peritonitis (TBP) is the most common clinical manifestation of abdominal TB, involving the peritoneum, mesentery, and omentum [6,7]. Despite the availability of various tools for the diagnosis of abdominal TB, the current approach involves using a combination of clinical, radiologic, endoscopic, microbiologic, histologic, and molecular techniques [8]. This is primarily related to the low sensitivity of microbiological tools for diagnosis. TBP may present in a variable manner with features like fever, abdominal distension, pain, loss of weight, and ascites. It is pertinent to note that the diagnosis of TBP is often difficult as microbiological positivity in ascitic fluid is uncommon. The diagnosis often rests on the ascitic adenosine deaminase with a level of >39 U/L being considered fairly sensitive and specific for TBP. Molecular tests, like the Xpert MTB/RIF, have low sensitivity for the diagnosis of TBP [9]. Therefore, it is of the utmost importance to distinguish TBP from peritoneal carcinomatosis, which it mimics closely.

Peritoneal carcinomatosis (PC) is an invasion of the serous membrane lining the abdominal cavity by malignant cells. PC can be a result of metastasis from intra-abdominal or extra-abdominal malignancies. Abdominal sources of metastasis commonly include the ovaries, large bowel, stomach, and pancreaticobiliary; extra-abdominal sources commonly include the breast, lung, thyroid, and lymphoma [10]. Diagnosis of PC symbolized a poor prognosis due to the advanced stage of malignancy and limited treatment modalities available in the past. Recent advances in the last few decades, like cytoreductive surgery (CRS) which targets macroscopic disease, and hyperthermic intraperitoneal chemotherapy (HIPEC) which targets microscopic residual disease, have provided promising outcomes [11]. These techniques are aggressive and associated with high morbidity. Early and accurate diagnosis, as well as the timely initiation of therapy, is crucial for optimal efficacy.

One of the close mimics of PC is TBP. The diagnosis of TBP may be delayed due to the insidious and intersecting clinical presentation of abdominal pain, distension, intestinal obstruction, and fever [7,8,12]. However, when no clear suggestion of primary cancer in the ovary or any other organ is found from imaging, the differentiation between TBP and PC can be challenging. This is especially the case with overlapping imaging findings of diffuse infiltration of the peritoneum, omentum, or mesentery [13]. Furthermore, with no specific laboratory test to separate the two entities, the clear separation between the two often requires laparoscopy or histopathological confirmation.

Computed tomography with an intravenous contrast is frequently the imaging modality of choice and is used in the non-invasive evaluation of peritoneal involvement, due to its feasibility and availability. Cross-sectional imaging provides adequate visualizations of all pockets of the peritoneal cavity and subdiaphragmatic spaces, which may not be easy with diagnostic laparoscopy. A few studies have tried to differentiate TBP from PC using computed tomography, however significant overlap between the two persists [14,15,16]. The information provided by conventional radiological imaging is limited to the morphological anatomy, whereas molecular imaging provides additional information on physiological and pathological processes [17]. Imaging modalities like diffusion-weighted magnetic resonance imaging and fluorodeoxyglucose positron emission tomography (FDG PET) have improved the diagnostic performance compared to CT in differentiating PC from TBP [18]. Newer modalities like dual time point imaging and 68 Ga-FAPI-04 (fibroblast activation protein-specific inhibitor) PET/CT imaging have greatly improved the specificity for diagnosing malignant over inflammatory lesions [19,20]. The use of dual and delayed time point imaging is based on the differential changes in avidity related to the different levels of glucose-6-phosphatase in benign and malignant lesions. The FDG uptake increases with time in malignant lesions. Similarly, FAPI PET has a role in detecting malignancies within a fibroblast dominant microenvironment including PC, but its response in TBP is not known. However, the availability, cost, and required expertise limit the utility in resource-constrained economies. Image-guided peritoneal biopsies have provided better diagnostic yields, while laparoscopic biopsies are invasive as well as expensive. Therefore, we planned an observational study describing the clinical features and computed tomographic findings in TBP and PC to identify the features which could help in differentiating these two entities.

## 2. Materials and Methods

### 2.1. Study Design and Patients

We conducted an ambispective analysis of patients suspected as having tuberculous peritonitis or peritoneal carcinomatosis between January 2020 to December 2021 at a tertiary care center in North India. All consecutive patients presenting with ascites who were suspected to have peritoneal carcinomatosis or tuberculous peritonitis were considered for inclusion. We excluded patients with features of other etiological causes of ascites, e.g., those with chronic liver disease, chronic kidney disease, and heart failure. The study was approved by the institutional ethics committee vide letter number INT/IEC/2020/SPL-679 dated 30 May 2020. Some of the patients were included as part of an ongoing randomized trial that focused on the role of rolling over prior to paracentesis to improve the cytological yield [21]. Other patients were identified from our medical records. All patients provided written informed consent prior to inclusion and additional consent prior to any invasive procedure which was deemed as clinically relevant for the evaluation. Guidelines related to ethical human research including the Declaration of Helsinki and the Indian Council of Medical Research were followed.

### 2.2. Work Up and Follow-Up

The clinical features at the time of presentation included abdominal distension, abdominal pain, intestinal obstruction, a lump in the abdomen, and fever. History of loss of weight and past history of TB or malignancy were recorded. The patients underwent relevant evaluations guided by the radiological findings. Those with ascites underwent diagnostic paracentesis (blinded or radiology-guided). The samples were sent for cytological analysis for malignant cells, cytology (differential cell count), ascitic fluid Xpert MTB/RIF, ascitic adenosine deaminase, and the serum albumin ascites gradient. Cytological analysis for suspected peritoneal carcinomatosis was done thrice in patients where the initial evaluation did not yield a diagnosis. Those with an unclear diagnosis and having omental and/or peritoneal thickening were considered for an ultrasound-guided fine needle aspiration/biopsy for evaluation of the cause of the disease.

### 2.3. Definitions

The final clinical diagnosis was based on the gold standard as defined below. Peritoneal carcinomatosis (PC) was diagnosed on the basis of positive cytological findings on examination of the peritoneal fluid and/or fine needle aspiration from peritoneal/mesenteric/omental lesion and/or positive histopathological findings from surgical specimens [22]. The diagnosis of tuberculous peritonitis (TBP) was on the basis of positive cytological findings (caseating granuloma or granuloma) on fine needle aspiration from omentum/peritoneal lesions, elevated ascitic fluid adenosine deaminase (ADA) i.e., >39 U/L, microbiological positivity (culture or Xpert MTB/RIF) in ascitic fluid or response to antitubercular therapy (ATT) as evidenced by the disappearance of ascites within two months of initiation of ATT [8]. Some patients with an equivocal diagnosis (e.g., ADA > 30 but <39 U/L) were started on ATT and were considered to have TBP only in cases where objective evidence of a response to antitubercular therapy was documented in the form of resolution of ascites.

### 2.4. CT Techniques and Analysis

All CT scans were performed using multidetector row CT scanners. The scans were performed following an intravenous injection of 80–100 mL of non-ionic iodinated contrast agent. The scans of the entire abdomen and pelvis were acquired in the portal venous phase (70–90 s from the start of contrast injection).

All computed tomography (CT) scans were reviewed by a gastrointestinal radiologist with 10 years of post-training experience and a nuclear imaging expert with 10 years’ experience (RK). The experts were aware of the research query, i.e., radiological discrimination of TBP and PC, but were not unaware of the clinical findings, ascitic workup, investigations or the diagnosis. A predesigned format was provided for the documentation of findings in all cases. Any discrepancies were sorted by discussion between the imaging experts.

Radiological features noted in all patients included the presence and density of ascites, and loculated ascites; lymphadenopathy; peritoneal, mesenteric and omental involvement; and bowel involvement. Additionally, the liver, spleen, pleural effusion, and adnexal involvement (in females) were noted.

We graded the ascites as mild, moderate and severe [23]. The attenuation of ascites was defined as high or low if the attenuation value was >10 or <10 HU, respectively. If lymphadenopathy was present, the site, presence of conglomeration, necrosis, and calcification were recorded. The presence of peritoneal thickening and peritoneal enhancement were noted. Peritoneal thickening was further categorized as smooth or nodular. The omental involvement was reported as smudged (hazy), nodular, or cake-like (soft tissue/sheet like mass). The presence and site of bowel thickening and dilatation were reported. Additionally, the clumping of bowel loops and presence of encapsulating membranes were assessed. Visceral organs like the liver and spleen were assessed for enlargement. Any focal lesions were also recorded. The attenuation of the liver relative to the spleen was also reported as high or low. The contour of these organs was assessed for scalloping caused by ascites. Mesenteric changes were assessed as the presence of any stranding or nodules. Lymphadenopathy was recorded as present or absent. Basic peritoneal anatomy, peritoneal thickening and omental involvement have been described elegantly through images previously; readers are guided to glance through for better understanding of metastatic patterns in cases of malignancy [24].

**Figure 1:**
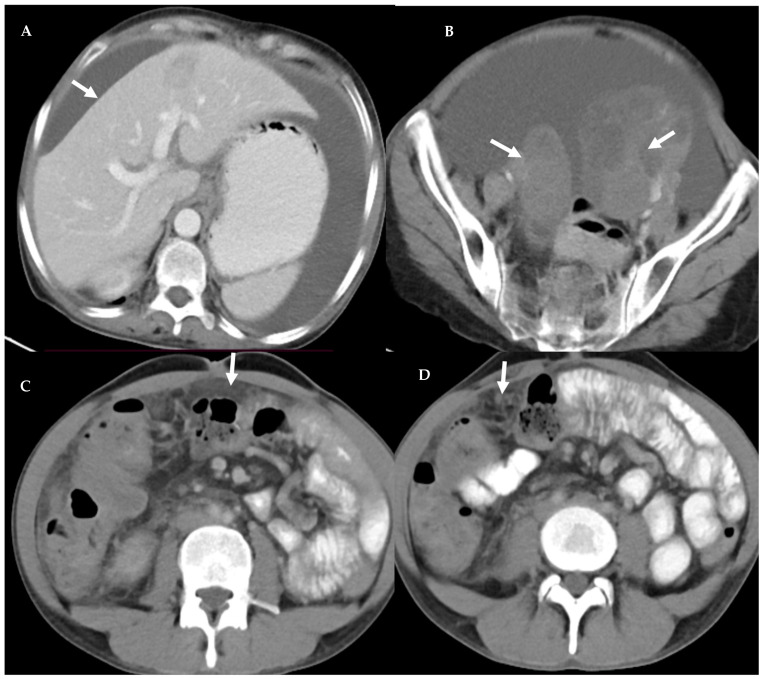
Peritoneal carcinomatosis. Axial contrast enhanced CT images in a 50-year-old lady. Ascites with scalloping of liver surface (arrow) can be seen (**A**). There are bilateral ovarian masses (arrow) (**B**). The peritoneum has a smudged appearance (arrow) (**C**). There are a few omental nodules (arrow) (**D**).

**Figure 2:**
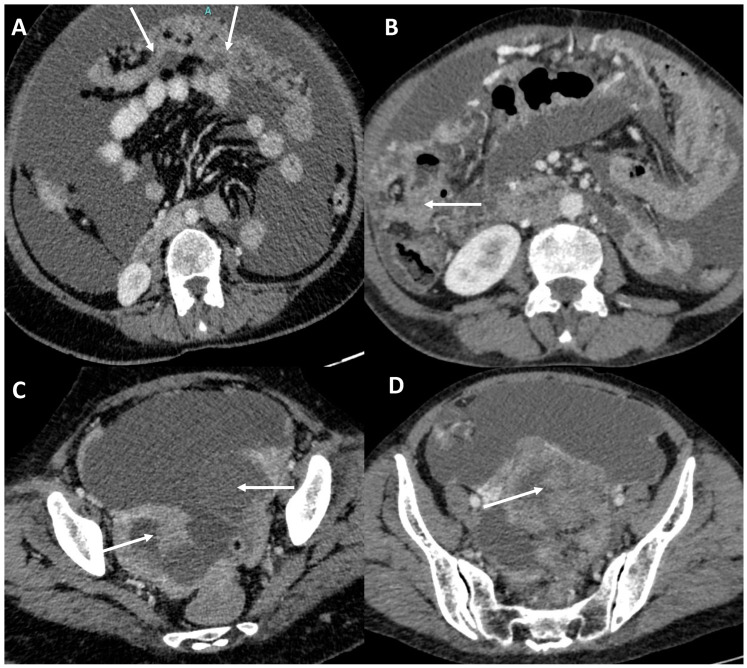
A 54-year-old female with peritoneal carcinomatosis. Axial contrast enhanced CT images show gross ascites with marked omental soft tissue thickening (“omental caking”, arrows, (**A**)) and serosal deposit along the ascending colon (arrow, (**B**)). Also note, bilateral adnexal masses (arrows, (**C**)) and large pelvic soft tissue deposit (arrow, (**D**)).

**Figure 3:**
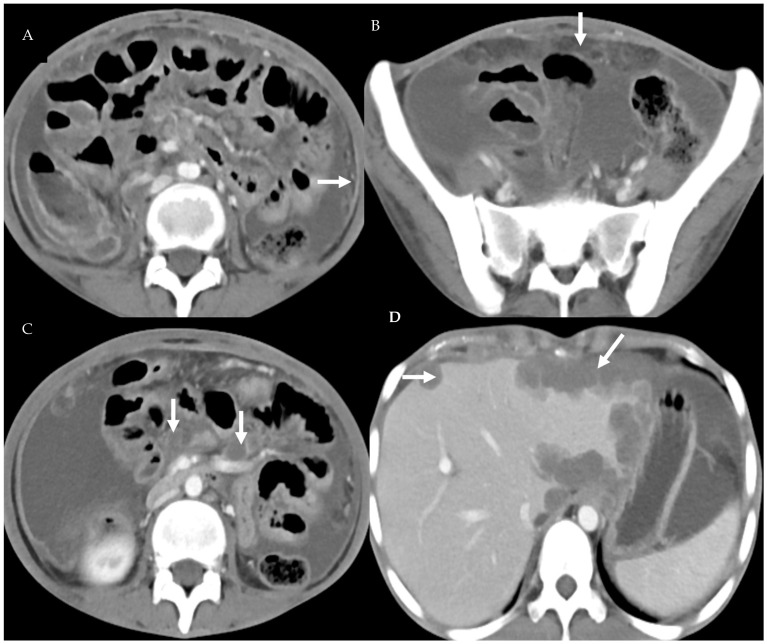
Peritoneal TB: Axial images of a 31-year-old lady with ascites show smooth peritoneal thickening and enhancement (arrow) (**A**). The omentum has a smudged appearance (arrow) (**B**). Few necrotic mesenteric lymph nodes can be seen (arrow) (**C**). There are multiple subcapsular liver lesions(arrow) (**D**).

**Figure 4:**
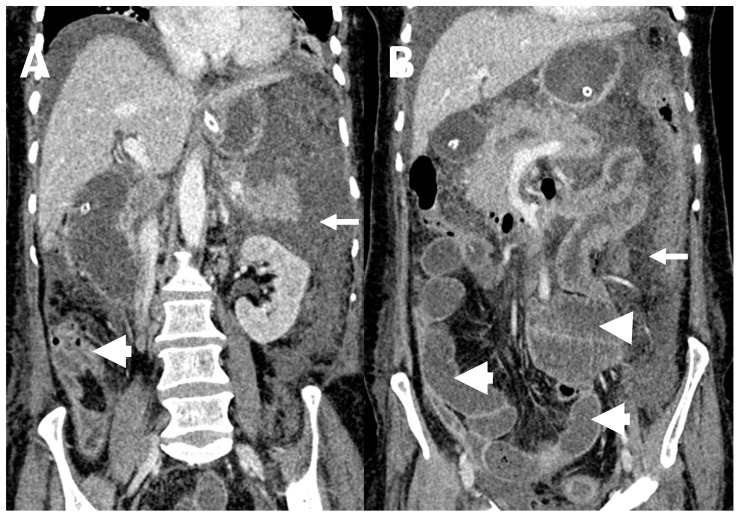
Peritoneal tuberculosis in a 44-year-old male. Coronal contrast enhanced CT images show moderate ascites (arrows, (**A**)) with diffuse omental stranding (arrow, (**B**)). Also note, associated small bowel dilatation (thick arrow, (**B**)) and mural thickening at the terminal ileum and ileocaecal junction (thick arrow, (**A**)).

Figure 1, Figure 2, Figure 3 and Figure 4 show typical radiological findings in patients of peritoneal carcinomatosis and peritoneal tuberculosis.

### 2.5. Statistical Analysis

All analyses were performed using Statistical Package for Social Sciences (SPSS) version 23.0. Continuous variables were summarized using the mean and standard deviation. Categorical variables were summarized as frequency and percentage. The Chi-square test was used to analyze the relationship between two categorical variables. Two-sided *p*-valves were reported and a *p*-value < 0.05 was considered statistically significant. The study population was grouped into two groups, i.e., TBP and PC. Continuous variables were compared using the Mann–Whitney U test, while categorical variables were compared using the Chi-square test. A regression analysis was performed to identify the independent predictors of peritoneal carcinomatosis.

## 3. Results

### 3.1. Patients

Around 110 patients were assessed for inclusion, but some were excluded for various reasons (4: clinical details not available, 17: diagnosis unclear or unproven). Therefore, a total of 89 patients were included in the study. There were 44 cases of tubercular peritonitis and 45 cases of peritoneal carcinomatosis. The mean age of the study group was 42.11 ± 16.39 years, and there were 35 (38.5%) males. Abdominal distension and loss of weight were the predominant complaints present in 73 (82%) and 70 (78.6%) patients, respectively. Overall, 58 (65.2%), 37 (41.6%), 18 (20.2%) and 11 (12.3%) patients had a history of pain in abdomen, fever, lump in abdomen and intestinal obstruction, respectively.

### 3.2. Clinical Differences

The median age of the study group was 31.5 years (IQR, 23.5–40) in TBP and 52 years (IQR, 46–61) in PC (Table 1). The median age in TBP was significantly lower as compared to PC (*p*-value < 0.001). The number of male patients was 19 (43.2%) in TBP and 16 (35.5%) in PC. The most common symptom in both TBP and PC patients was abdominal distension seen in 33 (75%) and 40 (89%), respectively (Table 1). Pain in the abdomen (34, 75.5%) and history of malignancy (17, 37.7%) were significantly associated with PC (*p* = 0.038 and *p* ≤ 0.001 respectively). Fever (32, 72.7%), history of TB (4, 9.1%), and history of weight loss (41, 93.2%) were significantly associated with TBP (*p* ≤ 0.001, *p* = 0.038 and *p* = 0.001) respectively. Table 1 shows the clinical features between the two groups.

### 3.3. Radiological Differences

The most common radiological finding was the presence of ascites which was noted in 42 (95.4%) and 42 (93.3%) TBP and PC patients, respectively (Table 2). Ascites was severe in 22 (52.3%) and 24 (57.14%) patients with TBP and PC, respectively (*p* = 0.664). Loculated ascites was noted more frequently in TBP (27, 64.3%) as compared to PC (9, 21.4%) patients (*p* ≤ 0.001). Abdominal lymphadenopathy was more common in PC (22, 48.8%) compared to TBP (13, 29.5%), but necrosis was found more in TBP (6, 46.1%) when compared to PC (8, 36.3%); however, both findings did not favor either condition and were not statistically different (*p* = 0.121 and *p* = 0.592). The conglomeration of lymph nodes (4, 30.7%) was significantly associated with TBP compared to PC (*p* = 0.038). Among the locations of lymphadenopathy, the mesenteric location (9, 69.2%) was significantly associated with TBP (*p* = 0.001). Overall bowel involvement was not significantly associated with either condition (*p* = 0.149), but the presence of membranes (58.8% vs. 0%, *p* = 0.001) and dilation of bowel loops (58.8% vs. 0, *p* = 0.001) were reported significantly more often in patients with TBP. Involvement of the liver in the form of reduced attenuation (11.4% vs. 37.7%, *p* = 0.004) and presence of focal lesion(s) (25% vs. 51.1%, *p* = 0.011) were noted significantly more in PC. Splenomegaly was significantly associated with TBP (6, 13.6%) compared to PC (*p* = 0.010).

Visceral scalloping of both the liver (25% and 37.8%) and spleen (4.5% and 8.8%) were similar in tuberculous peritonitis and peritoneal carcinomatosis. The involvement of adnexa was similar between the females of both groups (57% and 37.9%). Table 2 shows the major radiological findings between the two groups.

### 3.4. Peritoneal Involvement

Peritoneal involvement was noted in 32 (72.7%) and 33 (73.3%) patients with TBP and PC, respectively (Table 3). Peritoneal thickening was symmetric in 23 (71.9%) and asymmetric in 9 (28.1%) patients with TBP. Peritoneal thickening was symmetric in 20 (60.6%) and asymmetric in 13 (39.4%) patients with PC. Omental involvement was seen in 32 (72.7%) and 28 (62.2%) patients with TBP and PC, respectively. The most common omental involvement was a smudged appearance in both TBP (22, 68.7%) and PC (16, 57.1%). Nodular involvement of the omentum was significantly associated with PC as compared to TBP (14.3% vs. 0%, *p* = 0.029).

Mesenteric involvement was noted in 35 (79.5%) and 29 (64.4%) patients with TBP and PC, respectively (Table 3). Mesenteric stranding or nodularity were not significantly associated with either condition (*p* = 0.095 and *p* = 0.390). Table 3 shows the differences in both groups as per CT findings of the peritoneal structures.

### 3.5. Predictors of Peritoneal Carcinomatosis

For the bivariate logistic regression, we entered items like age, gender and those with a significance level of *p* < 0.10. These included fever, abdominal pain, distension, loss of weight, loculated ascites, mesenteric stranding, omental nodularity, conglomerate lymph nodes, presence of an encapsulating membrane around bowel, dilated bowel loops, hepatic attenuation, focal liver lesions, and splenomegaly. Using a forward logistic regression method, the factors which were independent predictors were age (OR: 1.102, 95% CI: 1.042–1.165, *p* = 0.001), fever (OR: 0.04, 95% CI: 0.007–0.22, *p* < 0.001), loculated ascites (OR: 0.060, 95% CI: 0.012–0.315, *p* = 0.001).

The sensitivity, specificity, positive predictive value, negative predictive value, and diagnostic accuracy of significant radiological variables are depicted in Table 4.

## 4. Discussion

It is challenging to recognize differentiation between TBP and PC early because of its insidious nature and intersecting symptoms. However, it is obligatory to provide an early diagnosis to avoid morbidity and provide definitive therapy. Contrast-enhanced CT is the imaging modality of choice for differentiation between TBP and PC, due to its widespread availability [25]. There are no characteristic or pathognomonic imaging findings for either PC or TBP, but clinical findings and the demographic origin of the patient, in conjunction with imaging findings may be indicative of a probable diagnosis. This study revealed overlapping findings between the two, but suggested clinical findings in conjunction with imaging features may be suggestive of the leading diagnosis. Clinical findings like young age, fever, and prior history of TB point towards TBP as the leading diagnosis. Similarly, radiological findings like loculated ascites, conglomerated lymph nodes, splenomegaly, and bowel involvement favored TBP; whereas nodular omental involvement and focal liver lesions favored PC.

Conceptually, symmetry or asymmetry of mesenteric and omental involvement is governed by the route of pathogenic spread in TBP and PC. In TBP, the hematogenous route of spread results in a uniform distribution, and in PC, asymmetry is the result of metastatic implants through ascitic fluid movement, which are dictated by the anatomical features of the abdominal compartment, the negative pressure of subdiaphragmatic spaces, intestinal peristalsis, and gravity [26,27]. Multiple studies have compared the radiological findings in the past and inconsistent results have suggested considerable overlap. Contrast-enhanced CT can elucidate various characteristics like ascites, pattern of peritoneal, mesenteric, omental involvement, and lymph node and visceral organ changes.

The diagnostic sensitivity and specificity of CT for diagnosing TBP have been reported to be around 65% and 90%, respectively [14,15]. Similarly for PC, the diagnostic sensitivity and specificity have been reported to be around 75% and 90% [14,15]. The landmark paper by Ha et al. first reported on the utility of abdominal CT in the discrimination of TBP and PC [14]. They reported mesenteric changes like thickening and macro nodules (>5 mm); and omental changes like a smudged appearance and presence of an omental line to be significantly associated with TBP. Irregular omental infiltration was significantly associated with PC, but omental caking was noted to be statistically similar between the two. They suggested a model which included mesenteric macro nodules, presence of an omental line, irregular infiltrated omentum, and splenic abnormalities for predicting the underlying diagnosis. In another recent study from South Asia, the presence of peritoneal macro nodules, smooth omentum, calcified lymph nodes, splenomegaly, and high-density ascites suggested the diagnosis of TBP, while PC was suggested by the presence of omental irregularity [15]. This study suggested that CT may be better in patients over 40 years for diagnostic discrimination because of the variability of imaging findings in young people. Another work by Charoensak et al. suggested that ascites, loculated ascites, smooth peritoneal thickening, a smudged omentum, mesenteric abnormalities, and smaller lymph nodes were suggestive of TBP. On the other hand, irregular peritoneal thickening, peritoneal nodules, omental caking or nodularity were more suggestive of PC [28]. In a smaller study by Rodríguez E et al. nodular implants and irregular peritoneal thickening were suggestive of PC [29]. In another study by Kang et al. which primarily focused on the value of acidic adenosine deaminase, the authors reported while smooth peritoneal involvement and mesenteric involvement were more suggestive of PTB, irregular or nodular thickening of the peritoneum suggested an underlying diagnosis of PC [30]. However, the most common CT findings noted with TBP are ascites, smooth peritoneal thickening, mesenteric involvement, and omental thickening. This study confirmed the results of previous studies where ascites have been found in the majority of patients with TBP and PC [29]. A systematic review which included 6 studies with 656 patients, where 262 patients had TBP and 394 patients were diagnosed with PC was conducted. Among 17 features that were studied for diagnostic accuracy, smooth peritoneal thickening showed the highest diagnostic accuracy with a specificity of 84% and sensitivity of 60% with AUC of 0.83 for the diagnosis of TBP [31]. The omental involvement pattern (nodular, smudged or cake-like), which has been commonly studied in most of the studies, was found to have low diagnostic yield. Contrary to this, in a study by Ramanan et al., which purely focused on the omental rim sign, the authors suggested that this sign on contrast-enhanced CT depicting a uniformly thick enhancing peripheral outline of the omentum in the venous phase was specific and sensitive for TB peritonitis [16] However, when the “omental line” finding by Ha et al. and the “omental rim” sign described by Ramanan et al. were combined, the pooled specificity and sensitivity of this finding for discriminating PTB from PC was 96% and 67%, respectively [31]. Similarly, lymph node necrosis and calcifications, and mesenteric macro nodules were fairly specific for TB, but showed poor sensitivity. Although ascites have been commonly found in both conditions, the density and presence of loculation yielded a poor diagnostic accuracy because of poor sensitivity and specificity [31]. A summary of these findings suggests that computed tomography may have some role in suggesting the underlying diagnosis, but is not diagnostic in most cases.

Clinical features can often guide clinicians in making a provisional diagnosis in the case of diffuse peritoneal disease. Patients with TBP are younger and have more inflammatory loads manifesting as a fever as compared to PC. This study confirmed the findings of previous studies where patients with TBP were younger and presented more commonly with a fever [32,33]. History of TB in the past or family history have been noted in 5–20% of the patients with active TB [5]. Approximately 10% of the patients in this study had a past history of TB, confirming similar findings from previous studies. Symptoms like abdominal distension, pain in abdomen, and loss of weight were present in the majority of our patients, similar to previously reported large studies; caution should be exercised in diagnosis based on these findings due to their non-specificity [15]. In a study that included multiple centers which evaluated the clinical characteristics and CT findings to discriminate between TBP and PC, they found that younger age, presence of fever, and night sweats were the clinical features suggestive of TB. On CT, the presence of an omental rim, and calcified or enhancing lymph nodes suggested TB; whereas omental caking, irregular peritoneal thickening or the presence of nodules, visceral scalloping, and a larger amount of ascites suggested PC. The authors suggested that a model including these parameters had an AUC of 0.914 in the discrimination of the two diseases [33].

The present study had a few limitations. Firstly, it was a single-center study, with relatively small numbers in both of the groups. However, the study also has strengths including a good follow-up rate with a clear diagnosis and blinding of the radiologist to the underlying diagnosis or clinical information. Further, the results were reported by two radiologists and the final opinion was based on consensus.

## 5. Conclusions

Most of the findings analyzed from CT overlap in both diseases. While loculated ascites, conglomerated lymph nodes, bowel dilatation, the presence of an encapsulating membrane around the bowel, and splenomegaly suggested TBP; focal liver lesions, hepatic attenuation, and omental nodularity suggested PC. None of these findings were specific for a particular diagnosis. The use of a combination of clinical features and radiological findings may suggest the underlying diagnosis but none of these features is specific to the underlying condition.

## Figures and Tables

**Table 1:** Baseline characteristics of tuberculous peritonitis and peritoneal carcinomatosis patients.

S. No	Character	Tuberculous Peritonitis (n-44)	Peritoneal Carcinomatosis (n-45)	*p* Value
1.	Age (yrs) (Median, IQR)	31.5 (23.5–40)	52 (46–61)	<0.001
2.	Male (%)	19 (43.2%)	16 (35.5%)	0.461
3.	Distension	33 (75%)	40 (88.8%)	0.088
4.	Pain in abdomen	24 (54.5%)	34 (75.5%)	0.038
5.	History of intestinal obstruction	8 (18.2%)	3 (6.6%)	0.099
6.	Fever	32 (72.7%)	5 (11.1%)	<0.001
7.	Lump in abdomen	8 (18.2%)	10 (22.3%)	0.635
8.	Loss of weight	41 (93.2%)	29 (64.4%)	0.001
9.	Past history of TB	4 (9.1%)	0	0.038
10.	History of malignancy	0	17 (37.7%)	<0.001

**Table 2:** Radiological features of TBP and PC.

S. No	Character		Tuberculous Peritonitis (n-44)	Peritoneal Carcinomatosis (n-45)	*p* Value
1.	Ascites		42 (95.4%)	42 (93.3%)	0.664
	Quantity	Mild to moderate	20(47.6%)	18(42.8%)	0.661
		Severe	22(52.3%)	24(57.14%)	0.661
2.	Loculated ascites		27 (64.3%)	9 (21.4%)	<0.001
3.	Low ascitic attenuation		23 (54.7%)	27 (64.2%)	0.373
4.	Lymphadenopathy		13 (29.5%)	22 (48.8%)	0.121
	Mean size in cm Lymph node necrosis		1.37	1.32	-
			6 (46.1%)	8 (36.3%)	0.592
	Conglomeration		4 (30.7%)	0	0.038
	Location	Periportal	3 (23.1%)	10 (45.4%)	0.220
		Mesenteric	9 (69.2%)	4 (18.2%)	0.001
		Retroperitoneal	0	3 (13.5%)	0.173
		Para aortic	1 (7.6%)	3 (13.5%)	0.623
		Peri gastric	0	2 (9.1%)	0.273
		Peri pancreatic	0	1 (4.5%)	0.445
5.	Bowel involvement		17 (38.6%)	11 (24.4%)	0.149
	Bowel characters	Clumping	12 (70.5%)	6 (54.5%)	0.102
		Membrane	10 (58.8%)	0	0.001
		Dilation	10 (58.8%)	0	0.001
6.	Liver involvement		13 (29.5%)	26 (57.8%)	0.007
		Reduced attenuation	5 (11.4%)	17 (37.7%)	0.004
		Scalloping	11 (25%)	17 (37.8%)	0.194
		Focal	11 (25%)	23 (51.1%)	0.011
7.	Splenomegaly		6 (13.6%)	0	0.010
	Spleen	SOL	4 (9.1%)	1 (2.2%)	0.159
		Scalloping	2 (4.5%)	4 (8.8%)	0.414
8.	Pleural effusion		23 (52.3%)	21 (46.7%)	0.597
9.	Adnexal involvement		14 (56%)	11 (37.9%)	0.184

**Table 3:** Radiological involvement of mesentery, peritoneum, and omentum in TBP and PC.

S. No	Character		Tuberculous Peritonitis (TBP) (n-44)	Peritoneal Carcinomatosis (PC) (n-45)	*p* Value
1.	Mesentery	Changes	35 (79.5%)	29 (64.4%)	0.113
		Stranding	32 (91.4%)	27 (93.1%)	0.095
		Nodularity	10 (28.6%)	7 (24.2%)	0.390
2.	Peritoneal involvement		32 (72.7%)	33 (73.3%)	0.949
	Peritoneal enhancement		32 (100%)	33 (100%)	0.949
	Peritoneal thickening type	Symmetric	23 (71.9%)	20 (60.6%)	
		Asymmetric	9 (28.1%)	13 (39.4%)	0.337
3.	Omental involvement		32 (72.7%)	28 (62.2%)	0.4
	Omental pattern	Nodular	0	4 (14.3%)	0.0269
		Caking	10 (31.3%)	8 (28.6%)	0.399
		Smudged	22 (68.7%)	16 (57.1%)	0.352

**Table 4:** Sensitivity, specificity, positive predictive value, negative predictive value, and diagnostic accuracy of significant radiological variables.

	S. No	Radiological Findings	TBP n-44	PC n-45	TP	FP	FN	TN	Sn (95% CI)	Sp (95% CI)	PPV (%)	NPV (%)	Diagnostic Accuracy (%)
**Favoring** **TBP**	1.	Loculated ascites	27	9	27	9	17	36	61 (45.50–75.64)	80 (65.40–90.42)	75	68	71
	2.	LN conglomeration	4	0	4	0	40	45	9 (2.53–21.67)	100 (92.13–100.0)	100	53	55
	3.	Bowel membrane	10	0	10	0	34	45	23 (11.47–37.84)	100 (92.13–100.0)	100	57	62
	4.	Bowel dilatation	10	0	10	0	34	45	23 (11.47–37.84)	100 (92.13–100.0)	100	57	62
	5.	Splenomegaly	6	0	6	0	38	45	14 (5.17–27.35)	100 (92.13–100.0)	100	54	57
	6.	Mesenteric LN	9	4	9	4	35	41	20 (9.80–35.30)	91 (78.78–97.52)	69	54	56
**Favoring** **PC**	7.	Focal liver lesions	11	23	23	11	22	33	51 (35.77–66.30)	75 (59.66–86.81)	68	60	63
	8.	Reduced liver attenuation	5	17	17	05	28	39	38 (23.77–53.46)	89 (75.44–96.21)	77	58	63
	9.	Omental nodularity	0	6	6	0	39	44	13 (5.05–26.79)	100 (91.96–100.0)	100	53	56

Sn—Sensitivity, Sp—Specificity, PPV—Positive predictive value, NPV—Negative predictive value. TP—True positive, FP—False positive, FN—False negative, TN—True negative.

## Data Availability

The datasets generated during and/or analyzed during the current study are available from the corresponding author upon reasonable request.
